# Manipulated Optical Absorption and Accompanied Photocurrent Using Magnetic Field in Charger Transfer Engineered C/ZnO Nanowires

**DOI:** 10.1002/gch2.202000025

**Published:** 2020-08-02

**Authors:** Jun‐Xiao Lin, Guan‐Xun Chen, Yen‐Fa Liao, Tzu‐Chun Hsu, Wei‐Jhong Chen, Kuo‐Yi Hung, Ting‐Yi Huang, Jiann‐Shing Lee, Zdenek Remes, Hua‐Shu Hsu

**Affiliations:** ^1^ Department of Applied Physics National Pingtung University No. 4–18 Minsheng Rd. Pingtung 90044 Taiwan; ^2^ National Synchrotron Radiation Research Center No. 101 Hsin‐Ann Road Hsinchu 30013 Taiwan; ^3^ Institute of Physics CAS Na Slovance 1999/2 Prague 182 21 Czech Republic

**Keywords:** charge transfer, magneto‐optical absorption, magneto‐photocurrent, spin‐polarized band engineering

## Abstract

The rarely explored, spin‐polarized band engineering, enables direct dynamic control of the magneto‐optical absorption (MOA) and associated magneto‐photocurrent (MPC) by a magnetic field, greatly enhancing the range of applicability of photosensitive semiconductor materials. It is demonstrated that large negative and positive MOA and MPC effects can be tuned alternately in amorphous carbon (***a‐C***)/ZnO nanowires by controlling the sp^2^/sp^3^ ratio of ***a‐C***. A sizeable enhancement of the MPC ratio (≈15%) appears at a relatively low magnetic field (≈0.2 T). Simulated two peaks spin‐polarized density of states is applied to explain that the alternate sign switching of the MOA is mainly related to the charge transfer between ZnO and C. The results indicate that the enhanced magnetic field performance of (***a‐C***)/ZnO nanowires may have applications in renewable energy‐related fields and tunable magneto‐photonics.

## Graphical abstract

The alternate magnetic‐field‐manipulated optical absorption (MOA) and associated photocurrent can be engineered by spin‐polarized band at room temperature. Simulated two peaks spin‐polarized density of states is applied to explain that the alternate sign switching of the MOA is mainly related to the charge transfer between ZnO and C.

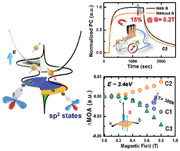

## 1 Introduction

The manipulation of optical absorption (OA) and the associated photocurrent (PC) can be a feasible approach to promote the use of photoinvolved energy applications^[^ ^1^, ^2^ ^]^ in fields such as optoelectronics and photonics;^[^ ^3^ ^]^ however, achieving increased participation of PC in the aforementioned processes is challenging. By obtaining delocalized electrons from photosensitive semiconductor materials, we can acquire a flow of photogenerated charge carriers due to the charge transition through optical absorption in accordance with the basic concept of the photoelectric effect.^[^ ^4^ ^]^ Static bandgap engineering strategies involve optical manipulations by doping^[^ ^5^ ^]^ or integrating metals to achieve the local surface plasmonic effect.^[^ ^6^, ^7^ ^]^ Moreover, bandgap engineering is generally regarded as a suitable strategy for promoting efficient PC harvesting. The effectiveness of this strategy has been examined through comprehensive experimental and theoretical calculations in metal‐oxide materials.^[^ ^8^, ^9^, ^10^ ^]^

By contrast, the dynamic modification of flowing PC has been successfully achieved using thermal,^[^ ^11^, ^12^ ^]^ force,^[^ ^13^ ^]^ and electric fields.^[^ ^14^, ^15^ ^]^ For dynamic modification, studies have focused on the noncontact approach, in which a magnetic field is used as an external driving force. Studies have used the Lorentz force to boost carrier transport^[^ ^16^, ^17^ ^]^ and optimized rapid recombination^[^ ^18^ ^]^ to enhance the PC response. Typically, the magnetic‐field‐controlled optical phenomena in nano‐heterostructures consisting of magnetic‐components arises from the interactions between magnetic moment of an electron and applied magnetic field.^[^ ^19^, ^20^ ^]^ As the functions underlying these potential applications are determined by the density of states (DOSs), it is important to elucidate interrelations between the band structure and the magnetic response.

A promising alternative to engineer spin‐polarized DOSs relies on the charge transfer between charge reservoirs and suppliers in many vacancy‐rich oxide nanostructures with a high unoccupied DOSs through Stoner excitation.^[^ ^21^, ^22^, ^23^ ^]^ Engineering spin‐polarized band introduces directly magnetic field to dynamic control the magneto‐optical absorption (MOA) and associated magneto‐photocurrent (MPC), highlighting their exploration for magnetic‐field‐manipulations on energy‐involved applications and photonics devices, which allows photosensitive semiconductor material to greatly enhance the range of applicability.

Amorphous carbon (***a‐C***) features a graphite‐like sp^2^ and diamond‐like sp^3^ hybridization with their ratio being modified with fabrication conditions.^[^ ^24^, ^25^ ^]^ In addition, we noticed that sp^2^ hybridization provides a high potential for facilitating charge transfer at hybrid nanostructures interfaces due to the wide vacancy states.^[^ ^26^, ^27^ ^]^ Therefore, the incorporation of ***a‐C*** into various micro and nanostructures offers a huge possibility of potential applications. For example, C/TiO_2_ with unique up converted photoluminescence have also been reported, which relies on efficient absorbing visible light in carbon layer.^[^ ^28^ ^]^ ZnO nanostructures have been demonstrated as suitable candidate for energy harvesting.^[^ ^29^ ^]^ ZnO nanostructures exhibit good semiconductor properties, wide bandgap and surface states serving as carrier‐transfer reservoirs. Based these properties, spin‐polarized band engineering seems to be an ideal strategy for manipulation of MOA and associated MPC between two reservoirs of ***a‐C*** and ZnO nanostructures.

Numerous MOA and MPC effects can be observed in the ***a‐C***/ZnO NWs. The alternate negative and positive MOA and MPC effects can be tuned by controlling the sp^2^/sp^3^ ratio. Compared to zero magnetic field, an enhancement of 15% was achieved at room temperature (RT) in the MPC ratio with a relatively low magnetic field (≈0.2 T). Simulated two peaks spin‐polarized density of states is applied to explain that the alternate sign switching of the MOA is mainly related to the charge transfer between ZnO and C. This result may be useful for achieving magnetic field enhanced performance in renewable energy‐related fields and future tunable photonic applications.

## 2 Results and Discussion

**Figure 1:**
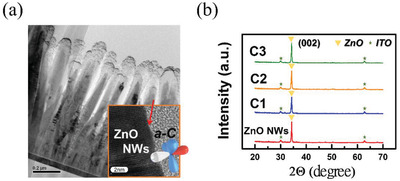
a) Cross‐sectional TEM image of C3. HRTEM images of C3 interfaces are presented in the inset. b) XRD patterns of the bare ZnO NWs and samples C1 to C3.

**Figure** **1**a displays the cross‐sectional transmission electron microscopy (TEM) image of ***a‐C***/ZnO NWs prepared with an ***a‐C*** sputtering power of 150 W (C3). The high‐resolution TEM (HRTEM) images of ***a‐C***/ZnO NW interfaces are illustrated in the inset. The images of ***a‐C***/ZnO NWs obtained with an ***a‐C*** sputtering power 50 W (C1) and 100 W (C2) are displayed in Figure S1a–c in the Supporting Information. Comparing ***a‐C***/ZnO NWs with bare ZnO NWs, we observe that the interfaces of the ***a‐C***/ZnO NWs were not destroyed through sputtering at different powers. In addition, the crystal structure and growth direction of the ZnO NWs were confirmed using X‐ray diffraction (XRD), which was performed using a Cu target in the X‐ray tube. Typical θ–2θ XRD spectra were obtained. No remarkable shifts in any ZnO diffraction peaks and peak broadening are observed for the ***a‐C***‐coated ZnO NWs, as displayed in Figure 1b. The XRD and HRTEM results indicate that the ***a‐C*** sputtering process did not noticeably affect the crystallinity of ZnO NWs.

**Figure 2:**
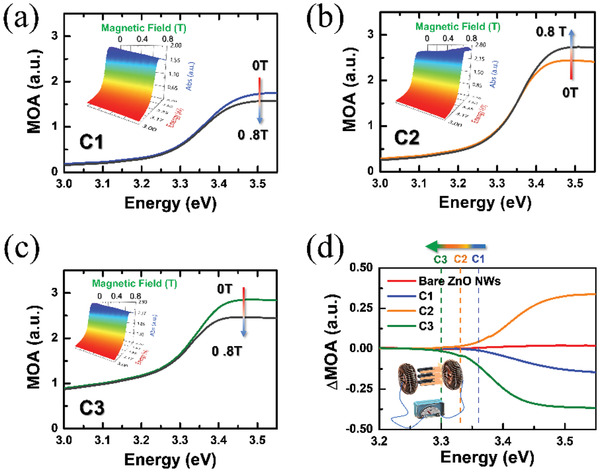
a–c) Optical absorption (OA) spectra of C1, C2, and C3 without and with a magnetic field (*B* = 0.8 T). d) The ΔMOA–*E* curves of all the samples. *a‐C*‐coated ZnO NWs exhibited a considerable MOA change after the application of a magnetic field.

The MOA spectra of C1, C2, and C3 measured at room temperature (300 K) without and with a magnetic field (*B* = 0.8 T) are illustrated in **Figure** **2**a–c. The applied external magnetic field was perpendicular to the specimen. Compared with the bare ZnO NWs, which indicated no obvious MOA change in the energy measurement region with and without a magnetic field (Figure S2, Supporting Information), the ***a‐C***‐coated ZnO NWs exhibited a considerable change in the MOA in the ultraviolet (UV) region with the applied magnetic field. The change was initiated from the onset of ZnO energy bandgap absorption (≈3.4 eV). Notably, for the C1 sample, the absorption change with increasing B was negative (called negative MOA). However, for the C2 sample, the absorption change with increasing B was positive (called positive MOA). When the ***a‐C*** sputtering power increased to 150 W (the C3 sample), the OA change became negative again. The alternate sign switching of the MOA effects could be exploited in our fabricated ***a‐C***/ZnO NWs. Figure 2d illustrates the energy‐dependent MOA change (ΔMOA) in all samples. ΔMOA can be defined as MOA(B)‐ MOA(0 T) for the ***a‐C***/ZnO NWs. Notably, the onset of ΔMOA shifted to low energy on increasing the ***a‐C*** sputtering power.

**Figure 3:**
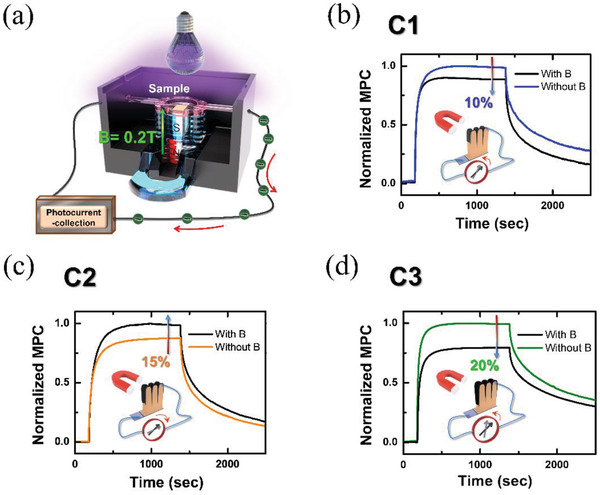
a) Detected MPC system when using a typical permanent magnet with *B* = 0.2 T. The specimen subjected to 18 mW UV light irradiation. b–d) Normalized MPC evolution for *a‐C*‐coated ZnO NWs at different sputtering powers. The MPC evolution is coincidentally consistent with the alternate sign switching of the MOA effect.

Typically, the PC varies when changing the MOA of light‐sensitive materials. Therefore, the MOA effect may be associated with MPC change due to the modified MOA caused by the application of a magnetic field. In order to meet the practical MPC applications, we took a typical permanent magnet (*B* = 0.2 T) as an external magnetic field source to detect PC change in our ***a‐C***/ZnO NWs. A schematic diagram of the experimental MPC measurements is displayed in **Figure** **3**a. A permanent magnet was used, and the specimen was subjected to 18 mW UV light irradiation (*E* ≈ 3.5 eV). In addition, the PC change with and without a relatively small magnetic field was measured under the same conditions. Figure 3b–d depicts the normalized MPC evolution for different ***a‐C***/ZnO NWs on 10 × 10 mm^2^ of indium–tin–oxide (ITO) conductive substrate. For C1, the MOA declined as expected with the application of a magnetic field. The MPC ratio, which indicates the change in the MPC under an applied magnetic field, is expressed as $\frac{I(0.2\;\text{T})-I(0\;\text{T})}{I(0\;\text{T})}\times 100\%$. The MPC ratio of C1 was approximately −10% at MPC saturation. By contrast, the PC of C2 increased when a magnetic field was applied, and the MPC ratio became approximately +15%. This finding is favorable for magneto‐photonics and energy conversion applications. Moreover, the MPC ratio of C3 switched to approximately −20% due to the negative MOA effect. However, bare ZnO NWs exhibited no obvious MPC ratio signal, as displayed in Figure S3 in the Supporting Information. Notably, our ***a‐C***/ZnO NWs exhibited the alternate sign switching of the MPC when a magnetic field was applied, which is consistent with the switching of MOA effects. The manipulated MPC effects can also be applied in the fields of energy conversion enhancement and tunable magneto‐photonics by using relatively small magnetic field.

**Figure 4:**
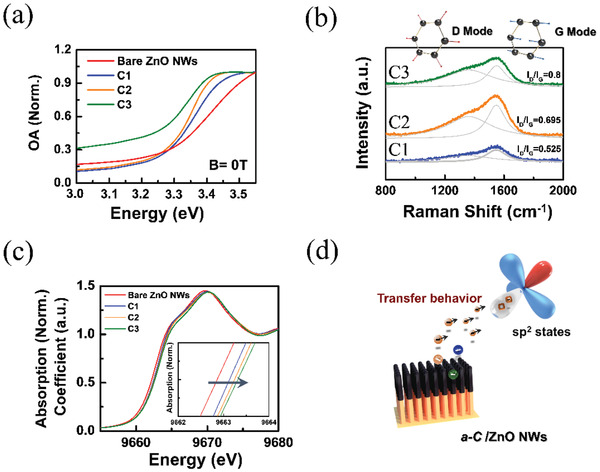
a) Normalized MOA spectra of the bare ZnO NWs and samples C1 to C3 at *B* = 0 T. The onset of absorption shifted to low energy with increasing *a‐C* sputtering power. b) Raman spectra of samples C1 to C3. The I(D)/I(G) ratio increased with increasing sputtering power. c) Normalized Zn K‐edge XANES spectra of different *a‐C*/ZnO NWs. d) Schematic diagram of electron transfer from the surfaces of ZnO NWs to *a‐C*.

The normalized MOA spectra for *B* = 0 T indicated that the onset of absorption shifted to low energy when increasing the *a‐C* sputtering power, as depicted in **Figure** **4**a. This implies that the *a‐C* coating can induce charge transfer between the *a‐C* and ZnO interfaces. The sp^2^ states of *a‐C* were expected to behave as vacancy states for the charge reservoir and transfer the charge from ZnO to C. Therefore, the ZnO absorption shifted to low energy. The charge transfer process is crucial for switching the MPC and MOA effects. Raman spectra and X‐ray absorption spectra (XAS) for the Zn K‐edge were obtained to gain additional insight into the charge transfer process. The relative intensity of the peaks of the Raman vibration modes for the G and D bands [I(D)/I(G)], which represents the sp^2^/sp^3^ ratio, is illustrated in Figure 4b. The sp^2^ states in our *a‐C*/ZnO NWs increased with increasing *a‐C* sputtering power, which is consistent with the findings of other studies.^[^ ^30^, ^31^ ^]^ In addition, XAS provided information on the electronic structure of the NWs, especially their valence states. The normalized Zn K‐edge X‐ray absorption near‐edge structure (XANES) spectra of different *a‐C*/ZnO NWs are displayed in Figure 4c. Typically, the half‐height energy of the XANES edge can be used to quantify the valence states of elements. The shifts in the half‐height energy of the Zn K‐edge XANES with increasing *a‐C* sputtering power can be associated with electron transfer from the surfaces of ZnO NWs to *a‐C*,^[^ ^32^, ^33^ ^]^ as displayed in the inset of Figure 4c. The results indicated that the Fermi level of the coated ZnO NWs decreased, as illustrated in Figure 4d.

**Figure 5:**
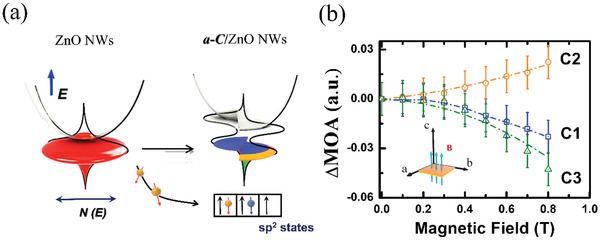
a) Schematic diagram of the proposed charge‐transfer‐induced spin‐polarized band. The charge transfer mechanism involves electron transfer from ZnO surfaces to *a‐C*, which increases the unoccupied *N(E)* and causes Stoner splitting. b) The ΔMOA(B) curves of the C1 to C3 samples at the onset energy of absorption. The dashed curves represent the results of the C1 to C3 samples fitted with a two‐peaks DOSs. The inset shows the measuring condition. The applied external magnetic field (*B*) is perpendicular to the specimen.

The high DOSs charge transfer induces spontaneous spin‐polarization of band structure. The formation of spin‐polarized band structures is a feasible approach for changing the physical properties of materials through Stoner excitation, as illustrated in Figure S4 in the Supporting Information. According to the MOA spectra, Raman spectra, and Zn K‐edge XANES results, the charge transfer occurred in our samples from the ZnO NW surfaces to *a‐C* and spin‐polarized bands were formed, as illustrated in **Figure** **5**a. The *a‐C* with sp^2^ states hybridizes with ZnO NWs defect band on the surface and result in a two peaks spin‐polarized band due to charge transfer.^[^ ^22^, ^23^ ^]^ In addition, because the unoccupied DOSs is proportional to the MOA, the manipulation of the two‐peaks band split by the applied magnetic field may alter the unoccupied DOSs, thereby changing the MOA and accompanied MPC effects. To further explore the relation between the two‐peaks spin‐polarized band and the observed MOA, we select a two‐peaks DOSs *N(E)* for one spin state

(1) $$\begin{array}{c} N\;\left(E\right)=\left(\frac{15}{4}\right)\;\left[8\frac{{\left(E-W\right)}^{2}}{W^{3}}\right]\left[1-\left[4\frac{{\left(E-W\right)}^{2}}{W^{2}}\right]\right],\;\text{for}\;0\leq E\leq 2W \end{array}$$

*N(E)* is usually defined as the DOSs at a specific energy, *W* is the defect bandwidth, and *E* is the onset position of the energy level sensitive to charge transfer extent. Because MOA can be considered as the total absorption of unoccupied DOSs, the change in the MOA by the applied magnetic field can be calculated as follows:

(2) $$\begin{array}{c} \Delta \text{MOA}=P\left\{N{\left(E\right)}_{↑}\left(E+m_{↑}B\right)+N{\left(E\right)}_{↓}\left(E-m_{↓}B\right)-2N\left(E\right)\right\} \end{array}$$

where *m* and *B* represent the spin moment and magnetic field, respectively. The major contribution to *m_↑_* and *m_↓_* is attributed to the spin up and spin down states, respectively; and *P* is the proportional constant. The magnetic‐field‐dependent ΔMOA(*B*) curves at the onset energy of absorption are displayed in Figure 5b. The fitting curves of the two‐peaks DOSs agree with the experimental ΔMOA(*B*) data, and the fitting constant is displayed in Figure S5 in the Supporting Information. The sequences of transformation were predicted as *E* = 0.091, 0.064, and 0.045. The decrease in *E* can be simulated as different electron transfer extent from the surfaces of ZnO NWs to ***a‐C*** with increasing sputtering power. The results further demonstrated that the charge transfer process plays an important role for alternate negative and positive MPC and MOA effects. Although the real band structure should be more complicated than that in our approximated model, the alternate sign switching of MPC and MOA effects observed in our ***a‐C***/ZnO NWs system can be qualitatively interpreted by considering the charge‐transfer‐modulated DOSs in the two‐peaks band. Our simple calculation based on DOSs may not actually capture the entire physics of the magnetic field effects on a‐C/ZnO NWs. A more explicit calculation of magnetic‐quantum effects in a realistic geometry is another alternative for understanding these effects. For example, some previous studies that directly incorporate the effect of magnetic/electric fields on NWs using Poisson–Schrodinger approaches could be also responsible for the prediction and explanation of the origin these effects.^[^ ^34^, ^35^, ^36^ ^]^ Although the detailed mechanism for the manipulation of the MOA and MPC for ***a‐C***/ZnO NWs under applied magnetic fields is still not fully understood yet, the new findings presented herein may suggest a possible mean for further renewable energy‐related fields applications.

## 3 Conclusions

In conclusion, alternate positive and negative MOA and accompanied MPC effects can be achieved in ***a‐C***/ZnO NW systems with different charge transfer extent which caused by sp^2^ states. The significant MOA change enables the tunability of magnetic field‐controlled photonic applications. The enhancement of MPC reached 15% under a relatively low magnetic field of 0.2 T. This result can be useful for magnetic‐field‐controlled energy conversion applications in the future. Our results indicated that spin‐polarized band engineering is a feasible method to achieve the alternate positive and negative MOA. The sp^2^ states of ***a‐C*** behaved as charge reservoirs and induced charge transfer from ZnO to C. The sp^2^/sp^3^ ratio modulated by different ***a‐C*** sputtering powers can cause different charge transfer. Therefore, the spin‐polarized band should be engineered. A two peaks spin‐polarized DOSs can be used to qualitatively interpret the observed MOA change. The engineering of the charge‐transfer‐induced band structures may vary from system to system. This study can motivate the further exploration of MOA and MPC effects in photosensitive semiconductor materials, which can enable renewable energy requirements to be met.

## 4 Experimental

##### Experimental Section

To improve the growth of ZnO NWs, ZnO seed layers were prepared using radio frequency (RF) sputtering with a ZnO target and power of 100 W on an ITO glass substrate. Hexamethylenetetramine (C_6_H_12_N_4_) and zinc nitrate hydrate (Zn(NO_3_)_2_∙6H_2_O) were dissolved in deionized water and heated to 80 °C for growing ZnO NWs at a potential of 0.7 V for 30 min (through electrochemical deposition).^[^ ^37^ ^]^ The samples C1 to C3 were prepared at a base pressure of ≈ 1 × 10^−6^ T and working pressure of approximately 2 × 10^−2^ T. Following electrochemical deposition, ***a‐C*** layers were further coated on the ZnO NWs by using RF sputtering powers 50 W (C1), 100 W (C2), and 150 W (C3). The corresponding thicknesses were ≈25 (C1), 50 (C2), and 80 (C3) nm.

##### Material Characterizations

The images of the C1 to C3 samples were obtained through HRTEM (JEOL‐JEM2010) with a standard focused ion beam, which can be used to perform Ga milling at 5–30 kV. XRD measurements were obtained using a Cu target for studying the structural properties of the samples. Furthermore, the MOA was obtained by using the JASCO 815 spectrometer. A 450‐W xenon lamp was used for irradiation, and the sample was set up in the middle of an electromagnet. To obtain the absolute spectra of the samples, the data were subtracted from the substrate and background signals. The MPC data were obtained using a two‐point electrode with a potential of 1 V in a Zahner Ennium electrochemical station. Raman spectroscopy was used to obtain information on the intensity of I(D)/I(G) for ***a‐C*** through a 532 nm Diode‐Pumped Solid‐State laser. The Zn K‐edge XANES spectra of bare ZnO NWs and the C1 to C3 specimens were collected using the BM‐Materials X‐ray study SP12B1 beamline at SPring‐8, Japan

## Conflict of Interest

The authors declare no conflict of interest.

## Author Contributions

J.‐X.L. designed and performed the experimental measurements and analysis. G.‐X.C., T.‐C.H., W.‐J.C., and K.‐Y.H. helped with the design of material synthesis protocols and measurements. Y.‐F.L. helped with the X‐ray absorption measurements. T.‐Y.H. and J.‐S.L. helped with the X‐ray diffraction measurements. Z.R. participated in the discussion of experimental results. J.‐X.L. and H.‐S.H. wrote the manuscript. H.‐S.H. conceived the idea and supervised the whole project.

## Supporting information

Supporting Information
