# Supplementary material for: Manipulated Optical Absorption and Accompanied Photocurrent Using Magnetic Field in Charger Transfer Engineered C/ZnO Nanowires
Source: Glob Chall. 2020 Aug 2;4(10):2000025. doi: 10.1002/gch2.202000025 (PMC7533846; doi:10.1002/gch2.202000025)
Supplement: Supplementary file 1 — Supporting Information [file GCH2-4-2000025-s001.pdf]

© 2020 Wiley-VCH GmbH

Supporting Information

for *Global Challenges,* DOI: 10.1002/gch2.202000025

Manipulated Optical Absorption and Accompanied Photocurrent Using Magnetic Field in Charger Transfer Engineered C/ZnO Nanowires

*Jun-Xiao Lin, Guan-Xun Chen, Yen-Fa Liao, Tzu-Chun Hsu, Wei-Jhong Chen, Kuo-Yi Hung, Ting-Yi Huang, Jiann-Shing Lee, Zdenek Remes, and Hua-Shu Hsu**

Copyright WILEY-VCH Verlag GmbH & Co. KGaA, 69469 Weinheim, Germany, 2020.

Supporting Information

Manipulated Optical Absorption and Accompanied Photocurrent Using Magnetic Field in Charger Transfer Engineered C/ZnO Nanowires

Jun-Xiao Lin,[1] *Guan-Xun Chen,*[1] *Yen-Fa Liao,*[2] *Tzu-Chun Hsu,*[1] *Wei-Jhong Chen,*[1] *Kuo-Yi Hung,*[1] *Ting-Yi Huang,*[1] *Jiann-Shing Lee,*[1] *Zdenek Remeš,*[3] *Hua-Shu Hsu*[*1]

J. X. Lin, G. X. Chen, , T. C. Hsu, W. J. Chen, K. Y. Hung, T. Y. Huang, Prof. J. S. Lee, Prof. H. S. Hsu

[1]Department of Applied Physics, National Pingtung University

No. 4-18 Minsheng Rd., Pingtung 90044, Taiwan

E-mail: hshsu@mail.nptu.edu.tw

Dr. Y. F. Liao

[2]National Synchrotron Radiation Research Center

No. 101 Hsin-Ann Road, Hsinchu, 30013, Taiwan

Prof. Z. Remeš

[3]Institute of Physics CAS

Na Slovance 1999/2, Praha 8, Czech Republic

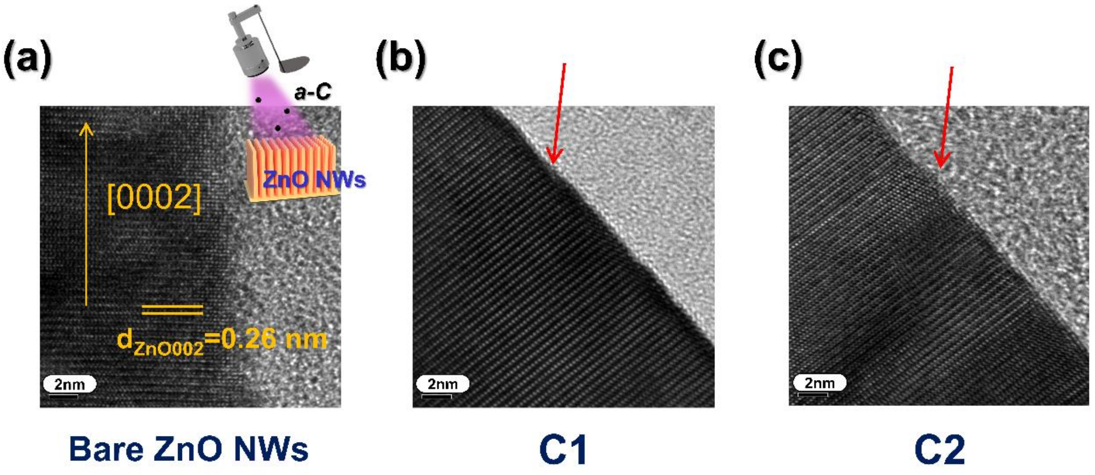

Figure S1. To determine the tunability of MOA effects, the microstructures of the ***a-C***/ZnO NWs were obtained through HRTEM (JEOL-JEM 2010). The bare ZnO NWs and ***a-C***-coated C1 and C2 samples are displayed in the figure. The ***a-C*** and ZnO NW surfaces were not destroyed by the sputtering process. The fringes along the axial direction of the ZnO NWs had a spacing of 0.26 nm, which can be attributed to the (002) planes of wurtzite ZnO. This implies that the [0002] direction is the growth direction of the ZnO NWs. The thickness of the ***a*-C** surfaces and the coated area increased with increasing ***a*-C** sputtering power.

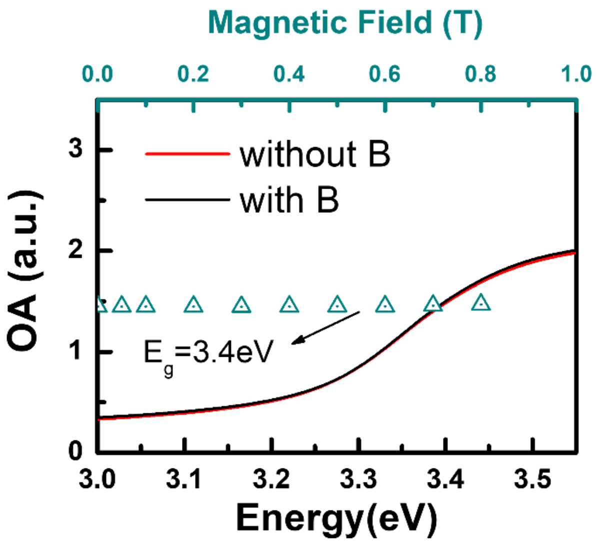

Figure S2. The bare ZnO NWs did not exhibit any OA change with increasing magnetic field. The magnetic-field-dependent MOA(B) indicated that the bare ZnO NWs were not affected by the applied magnetic field near the ZnO energy bandgap region.

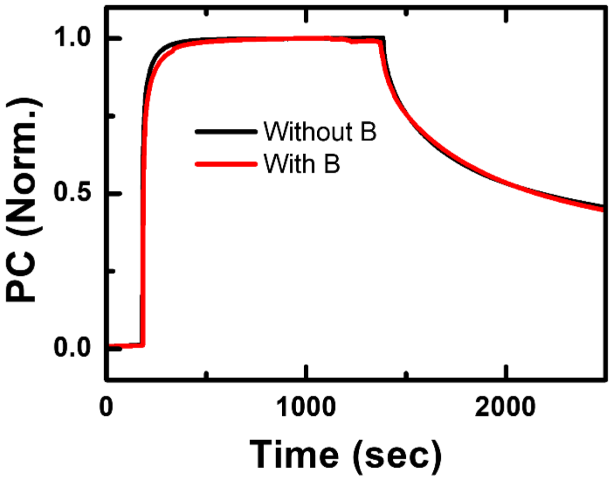

Figure S3. Under the same measuring conditions, the bare ZnO NWs did not exhibit distinguishable PC change when B = 0.2 T, which is consistent with the MOA effect for the bare ZnO NWs.

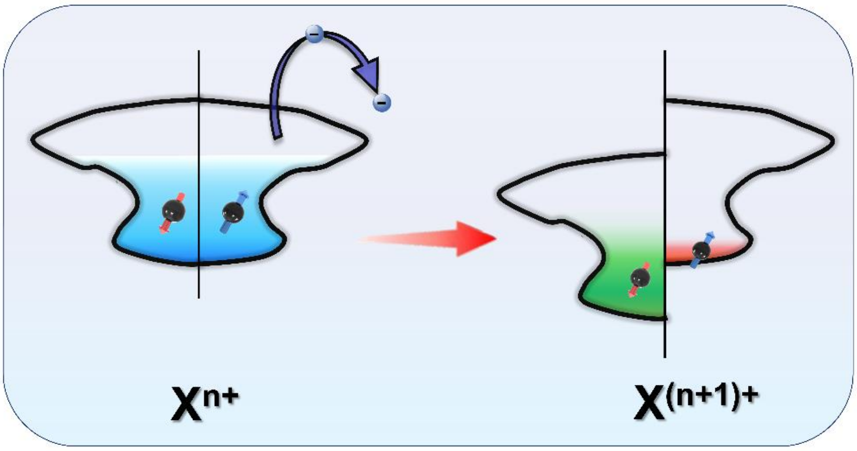

Figure S4. Schematic diagram of the idea underlying the charge transfer in the spin-polarized band. Surfaces with a high unoccupied DOSs are possible regions for charge transfer. When the electrons transfer from or to the reservoir, *N(E)* increases to induce a spin-polarized band, as observed in the standard Stoner excitation. In our study, the OA spectra and Zn K-edge XANES indicated that charge transfer occurred from the ZnO NW surfaces to ***a*-*C*** and spin-polarized bands were formed. This process corresponds to X^{n+} transforming to $X^{(n+1)+}$ [1].

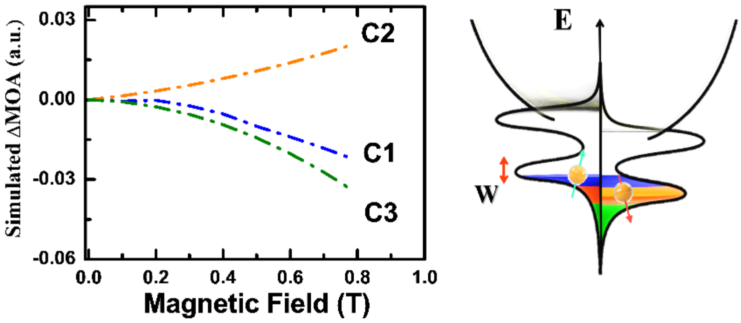

	C1	C2	C3
E	0.091	0.064	0.045
$m_\uparrow$	0.0078	0.003	0.0022
$m_\downarrow$	0.0081	0.0034	0.0023

Figure S5. Simulation of the ΔMOA–B dashed curves at the onset energy of optical absorption. The C1 to C3 samples were fitted by a two-peak DOSs, as depicted in the inset. The bandwidth was set as 0.075 eV and P was defined as 0.025 for this simulation. The two peaks of the spin-polarized band led to the transformation of MOA with changing the Fermi level. The sequences of transformation were predicted as E = 0.091, 0.064, and 0.045. The total m ($m_\uparrow + m_\downarrow$) also decrease with increasing of charge transfer, as demonstrated in the table.

References

[1] J. M. D. Coey, P. Stamenov, R. D. Gunning, M. Venkatesan, K. Paul, *New J. Phys*. **2010**, *12*, 053025.
